# Integrative Transcriptomic and Small RNA Analysis Uncovers Key Genes for Cold Resistance in Rice

**DOI:** 10.3390/genes16010038

**Published:** 2024-12-29

**Authors:** Fan Luo, Mengmeng Yin, Jianping Zhou, Xiaoli Zhou, Chunli Wang, Wenfeng Zhang, Lijuan Chen, Dongsun Lee

**Affiliations:** 1 Rice Research Institute, Yunnan Agricultural University, Kunming 650201, China; 17766749960@163.com (F.L.); nmengyin@126.com (M.Y.); 18228767665@163.com (X.Z.); wchunli1989@163.com (C.W.); 18683930792@163.com (W.Z.); 2 College of Agricultural Science, Panxi Crops Research and Utilization Key Laboratory of Sichuan Province, Xichang University, Xichang 615013, China; 3 School of Life Science and Technology, Center for Informational Biology, University of Electronic Science and Technology of China, Chengdu 610054, China; zhoujp@uestc.edu.cn; 4 The Key Laboratory for Crop Production and Smart Agriculture of Yunnan Province, Yunnan Agricultural University, Kunming 650201, China

**Keywords:** rice, chilling stress, miRNA sequencing, transcriptome sequencing, integrated analysis, miRNA-mRNA interaction

## Abstract

Background/Objectives: Cold stress is the main environmental factor that affects the growth and development of rice, leading to a decrease in its yield and quality. However, the molecular mechanism of rice’s low-temperature resistance remains incompletely understood. Methods: In this study, we conducted a joint analysis of miRNA and mRNA expression profiles in the cold-resistant material Yongning red rice and the cold-sensitive material B3 using high-throughput sequencing. Results: 194 differentially expressed miRNAs (DEMIs) and 14,671 differentially expressed mRNAs (DEMs) were identified. Among them, 19 DEMIs, including miR1437, miR1156, miR166, miR1861, and miR396_2 family members, showed opposite expression during the early or late stages of low-temperature treatment in two varieties, while 13 DEMIs were specifically expressed in Yongning red rice, indicating that these miRNAs are involved in rice’s resistance to low temperature. In the transcriptome analysis, 218 DEMs exhibited opposite expressions during the early or late stages of low-temperature treatment in two varieties. GO enrichment analysis indicated that these DEMs were enriched in biological processes such as a defense response to fungi, a defense response to bacteria, a plant-type cell wall modification, single-organism cellular processes, a response to chitin, and the regulation of a plant-type hypersensitive response, as well as in cellular components such as the apoplast, nucleus, vacuole, plasma membrane, and plasmodesma. Twenty-one genes were further selected as potential candidates for low-temperature resistance. The joint analysis of miRNA and mRNA expression profiles showed that 38 miRNAs corresponding to 39 target genes were candidate miRNA–mRNA pairs for low-temperature resistance. Conclusions: This study provides valuable resources for determining the changes in miRNA and mRNA expression profiles induced by low temperatures and enables the provision of valuable information for further investigating the molecular mechanisms of plant resistance to low temperatures and for the genetic improvement of cold-resistant varieties.

## 1. Introduction

Rice (*Oryza sativa* L.) is both a model plant and one that provides carbohydrate needs for over half of the world’s population [1]. As a tropical and subtropical crop, rice is susceptible to low-temperature stress. Under low-temperature conditions, rice may experience yellowing of leaves, wilting, tillering reduction, delayed heading, and spikelet sterility. In severe cases, it may even lead to plant death, ultimately resulting in a decrease in yield [2,3,4,5]. With the increase in rice cultivation area, rice planting is expanding from tropical and subtropical regions to high-altitude areas and cool summer areas, and the possibility of cold damage is increasing. Therefore, enhancing the cold tolerance of rice varieties is very important, indicating the need to explore the molecular mechanisms of rice cold tolerance.

MicroRNAs (miRNAs) are a class of noncoding endogenous small RNA in eukaryotes, typically ranging in length from 19 to 24 nucleotides (nt) [6,7]. It mainly regulates the expression level of its target gene mRNA through shear degradation, inhibition of translation, and chromatin remodeling (methylation). Numerous studies have shown that plant miRNAs play a crucial role in plant growth and development, epigenetics, and the response to biotic and abiotic stresses [7,8,9,10,11]. miR1432 negatively regulates abiotic stress tolerance by targeting *OsACAs* and affects plant growth and development [8]; meanwhile, another study found that it can also regulate drought tolerance by directly targeting the *CALMODULIN-LIKE2* gene in rice [12]. The overexpression of miR535 in rice can lead to low-temperature-induced cell death, excessive accumulation of ROS, and impaired osmotic regulation [13]. In maize, miR169s negatively regulates resistance to *bipolaris maydis* by affecting the SA-dependent signaling pathway and ROS accumulation [14]. miR397b participates in the regulation of root hair growth by targeting genes encoding reduced residual arabinose (RRA1/RRA2) in *Arabidopsis* (*Arabidopsis thaliana*) [15]. miR160, miR165, and miR166 are both involved in regulating the number, size, and weight of *Arabidopsis* seeds in a ROS-dependent manner [16].

The rapid development of sequencing technology and bioinformatics has led to the emergence of high-throughput sequencing as a prominent approach in the study of plant low-temperature defense mechanisms [17,18,19,20,21]. For example, through comparative transcriptome analysis, 795 DEMs related to low-temperature response were identified in the *indica* rice (*O. sativa* L. ssp. *indica*) TGMS 33S variety (cold-resistant material). Functional and enrichment analysis of these DEMs showed that the expression of genes related to nutritional reserve activity was significantly up-regulated in 33S, indicating that these genes play an important role in low-temperature response [17]. In *Camellia japonica* (Naidong), 4544 significant DEMs were identified through transcriptomics, and analysis revealed that transcription factors, lipid metabolism, and carbon metabolism play important roles in *Camellia japonica* (Naidong) resistance to low-temperature stress [18]. In Dongxiang wild rice, 16 significant DEMIs under low-temperature stress were identified through miRNA sequencing [19]. Among these miRNAs, miR408-5p was found to be significantly upregulated under low-temperature stress. The overexpression of miR408 has been shown to improve cold tolerance in rice [19,20]. Similarly, through miRNA sequencing, under low-temperature stress, 145 known and 876 novel miRNAs were identified; among them, miR164, MiR156, miR167, miR169, miR394, miR396, and miR398 were involved in response to low-temperature stress in maize [21].

Although there have been many independent reports on the low-temperature response transcriptome profiles of miRNAs and mRNAs, there are few reports on the joint analysis of miRNAs and mRNAs transcriptome profiles to reveal the mechanisms of low-temperature resistance. In order to further elucidate the molecular mechanism of rice response to low temperatures, this study applied high-throughput sequencing for the first time to combine analysis of the expression profiles of miRNAs and mRNAs in japonica rice (*O. sativa* L. ssp. *japonica*). The research results provide valuable resources for studying the specific expression of miRNA and mRNA in rice under low-temperature stress and help deepen our understanding of the miRNA–mRNA regulatory mechanism in rice under low-temperature stress.

## 2. Materials and Methods

### 2.1. Plant Materials and Stress Treatment

The *japonica* rice (*O. sativa* L. ssp. *japonica*) landrace Yongning red rice was provided by the Rice Research Institute of Yunnan Agricultural University. It is a high-altitude japonica rice distributed across Ninglang County, Lijiang City, Yunnan Province, China, which is the highest rice-producing area in the world at an altitude of 1800–2750 m. It has the characteristic of extremely low-temperature tolerance. *Japonica* rice (*O. sativa* L. ssp. *japonica*) strain B3 was provided by the Panxi Crops Research and Utilization Key Laboratory of Sichuan Province from Xichang University. It is sensitive to low temperatures. To investigate the low-temperature tolerance mechanism of Yongning red rice, Yongning red rice and B3 were used for a combined miRNA and transcriptome analysis. For analysis, three biological replicates were employed for each treatment, with 100 seedlings constituting the sample size for each replicate. Rice seeds were grown to the 3–4 leaf stage under a 12 h light/12 h dark cycle at 28 °C. The 3–4 leaf seedlings were then treated at 5 °C for a 12 h light/12 h dark cycle. Two rice lines were sampled at low temperatures for 0, 24, and 48 h. Five seedlings were randomly selected from each replicate to collect their root, stem, and leaf tissues. After washing away the soil, the tissues were immediately frozen in liquid nitrogen and stored at −80 °C until RNA extraction. The samples taken from the Yongning red rice line at 0, 24, and 48 h were labeled R0, Re, and Rl, respectively. The samples taken from the B3 line at 0, 24, and 48 h were labeled S0, Se, and Sl, respectively.

### 2.2. RNA Extraction

The total RNA was extracted from a mixture of rice roots, stems, and leaves using a Trizol reagent (Life Technologies, Carlsbad, CA, USA), according to the manufacturer’s instructions. The concentration and purity of the RNA were determined using a NanoDrop 2000 spectrophotometer (Thermo Fisher Scientific, Wilmington, DE, USA). RNA integrity was assessed using the RNA Nano 6000 Assay Kit on the Agilent Bioanalyzer 2100 system (Agilent Technologies, Palo Alto, CA, USA).

### 2.3. miRNA Library Construction and Sequencing

Small RNA libraries were constructed using the VAHTS™ Small RNA Library Prep Kit for Illumina (Vazyme, Nanjing, China) according to the manufacturer’s instructions. The small RNAs were first ligated with the 3′NEXTflex Adenylated Adapter, followed by the 5′ SR Adapter. After first-strand synthesis and PCR amplification, the final bands were purified and sequenced on Illumina’s NovaSeq 6000 platform. After sequencing, the reads were processed to remove any low-quality reads, reads containing greater than or equal to 10% unknown bases, adaptor sequences, and reads of <18 nt and >30 nt.

### 2.4. mRNA Library Construction and Sequencing

A total of 1 μg of RNA per sample was used to prepare the RNA libraries. Sequencing libraries were prepared using the Hieff NGS Ultima Dual-mode mRNA Library Prep Kit for Illumina (Yeasen, Shanghai, China), according to the manufacturer’s recommendations. The library quality was assessed on the Agilent Bioanalyzer 2100 system. Libraries were then sequenced on an Illumina NovaSeq platform to generate 150 bp paired-end reads according to the manufacturer’s instructions. Clean data were obtained by removing adapter-containing reads, ploy-N-containing reads, and low-quality reads from the raw data, and all downstream analyses were based on clean, high-quality data.

The clean reads were mapped to the rice reference genome (*Oryza_sativa*.MSU_v7.0) using Hisat2 tools [22]. Significant DEMs were identified by DESeq2 [23]. We used |log2(FC)| ≥ 1 and *p* ≤ 0.05 as thresholds to determine cold-induced DEMs.

### 2.5. Bioinformatics Analysis of Small RNA Sequencing Data

Clean reads were sequentially aligned with the Silva database, GtRNAdb, Repbase, and Rfam databases using Bowtie software. Unannotated reads containing miRNA sequences were obtained by filtering out noncoding RNAs (ncRNAs), such as snoRNA, tRNA, snRNA, rRNA, and repeat sequences. These unannotated reads were then compared with the rice reference genome (Oryza_sativa.MSU_v7.0) using Bowtie software to analyze their expression and distribution. To identify known miRNAs, alignment was performed with mature miRNA sequences from the reference genome and the miRBase (v22) database, including their downstream 5 nt and upstream 2 nt ranges. A mismatch of no more than one base was allowed for a sequence to be considered a known miRNA. For predicting novel miRNAs, miRDeep2 software was used to align the obtained reads to genomic positions to identify possible precursor sequences. Based on the distribution information of the reads on the precursor sequences (based on miRNA production characteristics, mathematics, star, loop) and precursor structure energy information (RNAfold randfold), Bayesian models were used for scoring, which facilitated the prediction of new miRNAs.

DEMIs were identified by DESeq2 with FC ≥ 1.5 (|Log2(FC)| ≥ 0.58) and *p* ≤ 0.05 as thresholds to determine cold-induced DEMIs. The target genes of the miRNAs were predicted using the TargetFinder program [24], and the sequences of the target genes were compared with the Swiss-Prot, NR, COG, GO, KOG, KEGG, and Pfam databases using BLAST tools.

### 2.6. qRT-PCR Analysis

Total RNA was extracted from mixed tissues of rice roots, stems, and leaves using a Trizol reagent (Life Technologies, CA, USA). Reverse transcription of miRNA and mRNA was performed using an M5 miRNA cDNA Synthesis Kit (Mei5bio, Beijing, China) and an M5 Super plus qPCR RT Kit with gDNA remover (Mei5bio, Beijing, China), respectively. Then, the M5 miRNA qPCR Assay Kit (Mei5bio, Beijing, China) and 2X M5 HiPer SYBR Premix EsTaq (Mei5bio, Beijing, China) were used for qRT-PCR analysis of miRNA and mRNA expression, using the CFX96 real-time PCR system (Bio-Rad, Berkeley, CA, USA). Three biological replicates were performed for each experiment. The expression of miRNAs and genes was calculated using the 2−ΔΔC (t) method [25]. Ubiquitin and U6 were used as internal reference genes for qRT-PCR analysis of mRNA and miRNA expression, respectively. Table S1 lists the primers used in qRT-PCR.

## 3. Results

### 3.1. Evaluation of Cold Tolerance in Rice Seedling Stage

**Figure 1:**
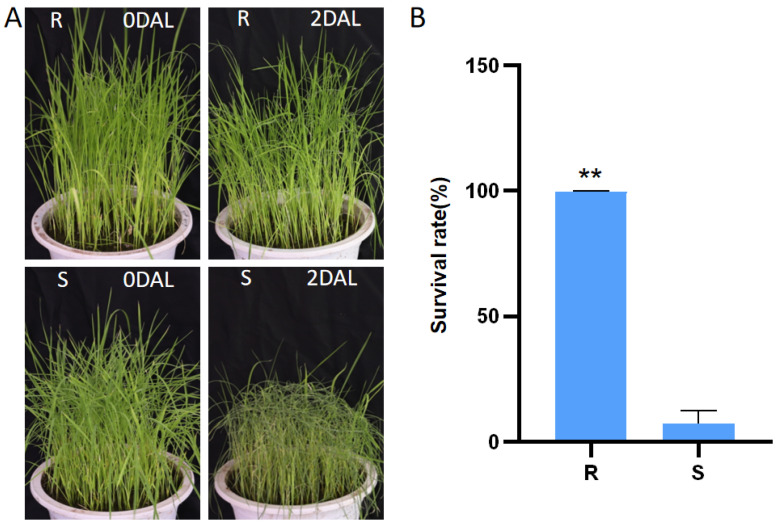
Evaluation of cold tolerance in the rice seedling stage of the Yongning red rice and B3. (**A**) Image of cold tolerance identification of Yongning red rice and B3 seedlings. (**B**) Survival rates of Yongning red rice and B3 plants after 2 days of cold treatment at 5 °C and a 5-day recovery period. R stands for Yongning red rice; S stands for B3; DAL, days after low-temperature treatment; ** for *p* < 0.01.

Yongning red rice exhibited a cold tolerance phenotype and had a higher survival rate compared to B3 (Figure 1). Therefore, Yongning red rice and B3 were selected for miRNA and mRNA sequencing before and after 24 and 48 h of low-temperature treatment. By comparing the miRNA and mRNA transcriptome profiles of Yongning red rice and B3, the mechanism of low-temperature tolerance in Yongning red rice was explored.

### 3.2. Small RNA Library Construction and Sequencing

Total raw reads of 18,762,842 to 56,372,907 were obtained in R0, Re, Rl, S0, Se and Sl libraries. After removing all low-quality reads, reads with unknown bases greater than or equal to 10%, adaptor sequences, and reads of <18 nt and >30 nt, a total of 11,722,741 to 21,890,454 clean reads remained in the 18 libraries, respectively (Table S2). A total of 831 miRNAs were obtained, of which 244 were novel miRNAs, accounting for 29.36%.

### 3.3. DEMIs Across Different Materials Before and After Low-Temperature Treatment

**Figure 2:**
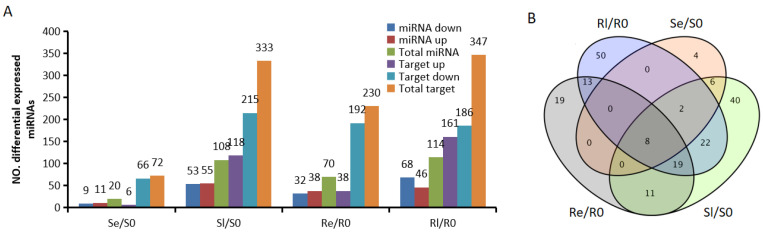
DEMIs in the comparisons. (**A**) Number of up- and downregulated miRNAs and target genes in comparing different low-temperature treatment periods (fold change > 1.5, *p* < 0.05). (**B**) Venn diagrams of the unique and common DEMIs.

After normalizing raw sequence reads, average normalized reads from three independent biological replicates were selected for further analysis. The expression levels of miRNAs were compared across the different groups. A total of 194 DEMIs were identified among the various cryogenic treatment stages with fold changes ≥ 1.5, *p* < 0.05 based on the average of three replicates (Figure 2B). In the early stages of low-temperature treatment, more DEMIs were observed in Yongning red rice (70) compared to B3 (20); however, in the late stages of low-temperature treatment, the number of DEMIs in Yongning red rice (114) and B3 (108) was not significantly different (Figure 2A), suggesting that many miRNAs involved in low-temperature regulation were not immediately expressed in B3.

### 3.4. Identification of miRNAs Related to Cold Tolerance

**Figure 3:**
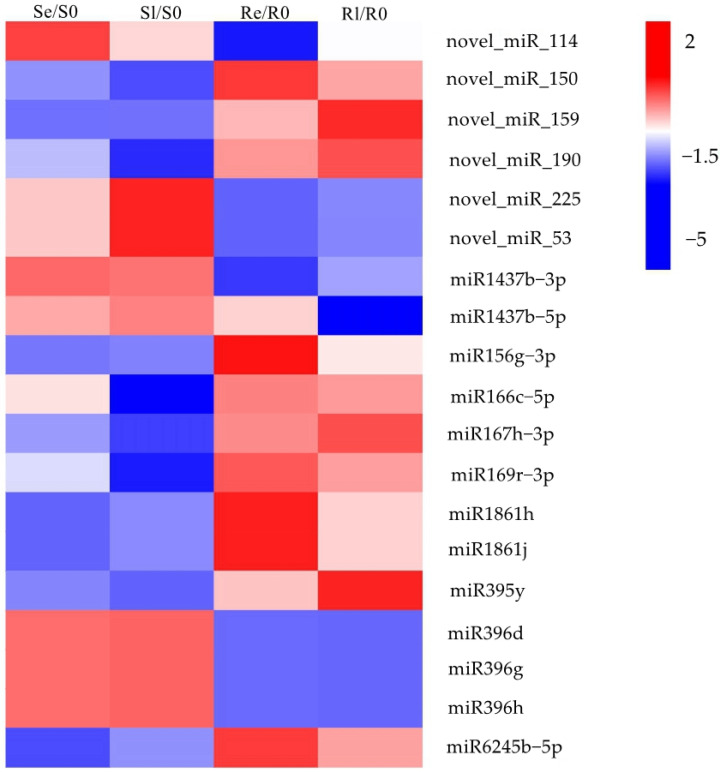
Heatmap of DEMIs were opposite expressed in two varieties after low-temperature treatment. The heatmap is constructed based on log2fold change values.

To identify miRNAs associated with plant chilling stress, Venn diagrams were used to visualize the DEMIs in early and late stages of low-temperature treatment for the two varieties (Se/S0, Sl/S0, Re/R0, and Rl/R0) (Figure 2B). In total, 69 DEMIs were expressed in Re/R0 or Rl/R0, and 13 were expressed specifically in Re/R0 and Rl/R0 (Figure 2B). Additionally, 19 DEMIs showed opposing expression patterns between the two varieties after low-temperature treatment (Figure 3). Of these, 6 were novel miRNAs, and 13 were known miRNAs, many of which belonged to known miRNA families, including miR1437, miRNA156, miR166, miR167_1, miR169_1, miR1861, miR395, miR396_2, and miR9783. Members of these miRNA families have been reported to play roles in various biological processes such as growth and development, flowering regulation, nutrient absorption, agricultural trait regulation, and responses to both biotic and abiotic stresses [26,27,28,29,30,31,32,33,34,35,36,37,38]. These DEMIs, which respond to chilling stress, may be involved in the regulation of low-temperature responses in rice.

### 3.5. General mRNA Expression Profiles

To analyze gene expression and profile all the targets of the DEMIs in response to cold tolerance, mRNA libraries were constructed. A total of 39,848,202 to 59,658,622 clean reads were sequenced from 18 mRNA libraries. After filtering out low-quality reads from the samples of Yongning red rice, 90.78–95.96% of the reads were mapped to 24,766–27,477 rice genes (Table S3). In the B3 replicates, 91.00–95.22% of reads were mapped to 26,335–28,109 rice genes (Table S3).

**Figure 4:**
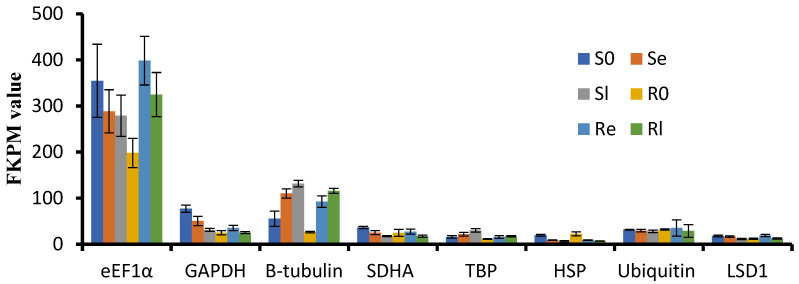
FKPM values of *GAPDH*, *SDHA*, *TBP*, *eEF1α*, *Ubiquitin*, *LSD1*, *β-tubulin*, and *HSP* from mRNA-sequencing data.

To select stable reference genes for qRT-PCR analysis during low-temperature treatment, we carefully selected eight commonly used reference genes: *SDHA* (*Os07g0424*), *LSD1* (Os12g41700), *TBP* (Os03g45410), *GAPDH* (Os02g38920), *β-tubulin* (*Os03g56810*), *HSP* (*Os03g31300*), *eEF1α* (*Os03g08020*) and *Ubiquitin* (*Os03g03920*). We evaluated the respective FPKM values of these genes from our RNA-seq data (Figure 4). Expression levels of *eEF1α*, *β-tubulin*, *GAPDH*, and *HSP* showed significant changes before and after low-temperature treatment, while *LSD1* and *TBP* were stable but relatively low in expression. Based on these findings, *Ubiquitin* was selected as the reference gene for qRT-PCR analysis.

### 3.6. DEMs in Response to Low-Temperature Treatment

**Figure 5:**
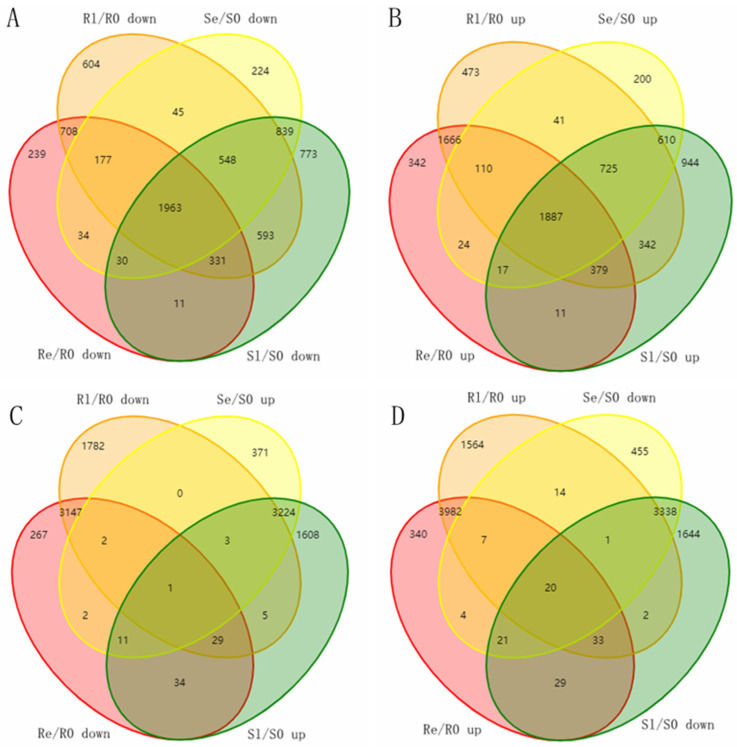
Venn diagram of the unique and shared DEMs. (**A**) Venn diagram of downregulated genes in Se/S0, Sl/S0, Re/R0, and Rl/R0. (**B**) Venn diagram of upregulated genes in Se/S0, Sl/S0, Re/R0, and Rl/R0. (**C**) Venn diagram of upregulated genes in Se/S0, Sl/S0, and downregulated genes in Re/R0 and Rl/R0. (**D**) Venn diagram of downregulated genes in Se/S0, Sl/S0, and upregulated genes in Re/R0 and Rl/R0. DEMs were screened using a threshold of fold change ≥ 2, *p* < 0.05.

A total of 14,671 DEMs (log 2 FC ≥ 2, *p* < 0.05) were detected between different low-temperature treatment stages (Table 1 and Figure 5). The DEMs in the Yongning red rice and B3 plants at various low-temperature treatment stages were hierarchically clustered. Across the four comparisons, the expression patterns of the DEMs were consistent, with the similar up- or downregulation observed (Figure S1).

During low-temperature treatment, the number of DEMs in Yongning red rice plants was consistently higher than that in B3. Notably, we found that the number of downregulated DEMs in Yongning red rice plants was consistently lower than that in B3, while more DEMs were upregulated in Yongning red rice plants compared to B3 plants (Table 1). These differential gene expression patterns between B3 and Yongning red rice likely contribute to the stronger cold tolerance observed in Yongning red rice.

### 3.7. Identification of Genes Associated with Cold Tolerance

To investigate the mechanism of rice’s resistance to low temperatures, sequencing data from Yongning red rice and B3 before and after low-temperature treatment were compared. Venn diagrams were used to analyze the potential genes related to low-temperature resistance in the DEMs of two rice genotypes, with a fold change (FC) ≥ 2 and *p* < 0.05. In Yongning red rice, 1551 unique DEMs were downregulated, and 2481 unique DEMs were upregulated after low-temperature treatment. In contrast, in the B3 plants, 1836 unique DEMs were downregulated, and 1754 unique DEMs were upregulated after treatment (Figure 5A,B). To better understand the function of these DEMs, GO enrichment analyses were carried out. In terms of biological processes, the downregulated genes in Yongning red rice and the upregulated genes in B3 plants were both enriched in processes such as pollen tube growth, defense response to fungus, defense response to bacteria, single-organism cellular process, response to chitin, regulation of plant-type hypersensitive response, response to cadmium ion, positive regulation of transcription, DNA-templated transcription, and protein targeting to membrane (Figure S2A,D). The upregulated genes in Yongning red rice and the downregulated genes in B3 were both enriched in the single-organism cellular process and the response to cadmium ion (Figure S2B,C). Among the molecular function GO terms, the most contrasting terms between the two rice varieties included quercetin 4′-O-glucosyltransferase activity, protein binding, peptide receptor activity, and ubiquitin protein ligase binding (Figure S2). Additionally, nine cellular components—apoplast, plasma membrane, Golgi apparatus, vacuole, chloroplast, nucleus, intracellular organelle part, plasmodesma, and plasma membrane—were enriched, indicating that these cellular components play a role in the low-temperature response (Figure S2).

**Figure 6:**
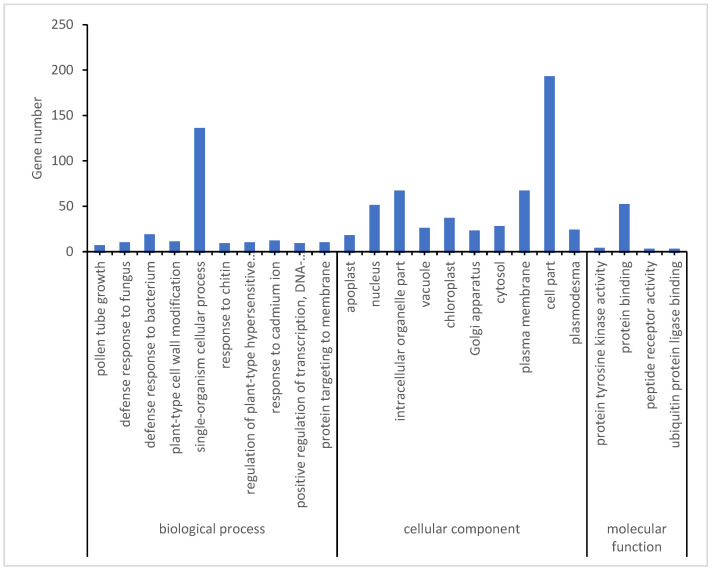
GO (Gene Ontology) analysis of DEMs with opposite expression in two materials during early or late stages of low-temperature treatment.

To further narrow down potential genes related to the low-temperature response, the opposite expression DEMs during the early and late stages of low-temperature treatment in two rice varieties was assessed. A total of 218 DEMs were identified in both Yongning red rice and B3 plants after low-temperature treatment, with 87 DEMs being downregulated in Yongning red rice plants and upregulated in B3, while 131 DEMs were upregulated in Yongning red rice and downregulated in B3 plants (Figure 5). GO enrichment analysis showed that these resistance-related genes were enriched in biological processes such as defense response to fungus, defense response to bacteria, plant-type cell wall modification, a single-organism cellular process, the response to chitin, and the regulation of a plant-type hypersensitive response. Cellular component enrichment included apoplast, nucleus, vacuole, plasma membrane, and plasmodesma (Figure 6). Finally, 21 genes were differentially expressed in both the Yongning red rice and the B3 after low-temperature treatment and were considered potential genes related to low-temperature resistance (Table 2). Among these, 20 genes were significantly upregulated in Yongning red rice and downregulated in B3 after treatment, while a single gene was downregulated in Yongning red rice and upregulated in B3. These DEMs were predominantly enriched in processes such as cellular processes, single-organism cellular processes, plant-type cell wall modification, stress response, metabolic processes, biological regulation, and cell wall organization. They were also enriched in cellular components such as the cell part, intracellular organelle part, nucleus, plasma membrane, and plasmodesma (Table 2).

### 3.8. Integrated Analysis of miRNA and mRNA Expression Profiles

In most instances, miRNAs negatively regulate target mRNAs by repressing translation or degrading mRNAs [39]. For correlating the identified miRNAs with their target genes, the psRNA target tool was used with the parameters fold change ≥ 1.5, *p* < 0.05 [40]. A total of 20, 105, 70, and 114 DEMIs showed significant negative correlations with 72, 333, 230, and 347 target mRNAs in Re/R0, Rl/R0, Se/S0, and Sl/S0, respectively (Figure 2A).

To identify potential miRNA–mRNA pairs associated with low-temperature response, 82 DEMIs from Re/R0 or Rl/R0 (Figure 2B) and 19 DEMIs from different low-temperature treatment stages (Figure 3) were selected and negatively correlated with 416 target mRNAs (Table S4). These miRNAs target different mRNAs at each stage of low-temperature treatment. For example, miR167h-3p was downregulated in Se/S0 and upregulated in Rl/R0. This miRNA negatively correlated with eight upregulated target genes in Se/S0 and two downregulated target genes in Rl/R0, respectively. However, most of these targets showed similar expression trends in both Yongning red rice and B3 after low-temperature treatment (Table S4). When these miRNAs and their corresponding targets were excluded, there were 38 miRNAs corresponding to 39 target genes that were differentially expressed in Re/R0 or Rl/R0, or that exhibited an opposite expression in Yongning red rice and B3 after low-temperature treatment. These were selected as potential miRNA–mRNA candidates related to low-temperature resistance (Table 3).

**Figure 7:**
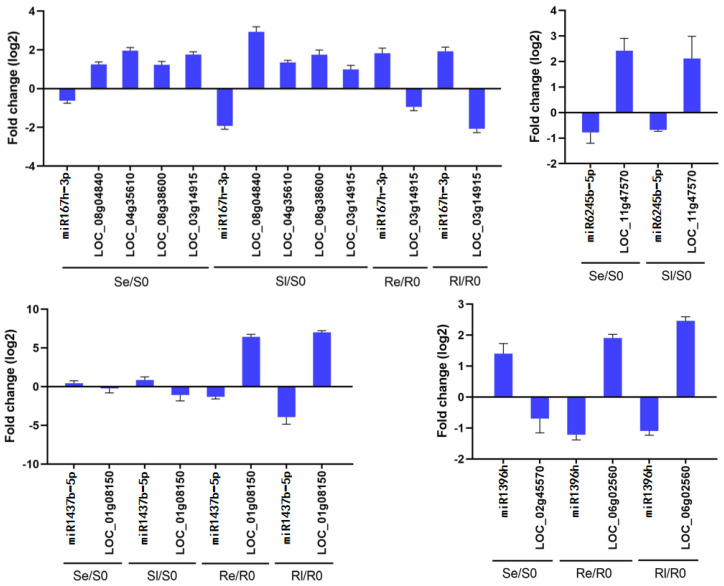
Comparison of the expression patterns of miRNAs and their target genes. Data are means ± SD of three independent biological experiments.

Four miRNAs and their target genes were chosen for qRT-PCR analysis to determine whether these miRNAs negatively regulate target gene expression. The results showed that miR1437b-5p negatively regulated *LOC_Os01g08150* in both Yongning red rice and B3, miR167h-3p negatively regulated *LOC_Os08g04840*, *LOC_Os04g35610*, *LOC_Os08g38600*, and *LOC_Os03g14915* in Se/S0 and Sl/S0, while it only negatively regulated *LOC_Os03g14915* in Re/R0 and Rl/R0. miR6245b-5p negatively regulated *LOC_Os11g47570* in both Se/S0 and Sl/S0, and miR396h negatively regulated *LOC_Os02g45570* in Se/S0, while it negatively regulated *LOC_Os06g02560* in Re/R0 and Rl/R0 (Figure 7). These results indicate that the qRT-PCR data are consistent with the sequencing results (Figure 7, Table 3).

## 4. Discussion

Few research reports have utilized a combined expression profile of miRNA and mRNA to analyze abiotic stress responses in plants, except for the study on the low-temperature regulation mechanism of the *CTS-12* locus in common wild rice (*Oryza rufipogon* Griff.) [41]. This study, for the first time, reports the integrated analysis of mRNA and miRNA expression profiles in japonica rice under chilling stress, thereby improving our understanding of the regulatory mechanisms involving miRNA–mRNA interactions under chilling stress in rice.

By comparing the miRNA expression of Yongning red rice and B3 before and after low-temperature treatment, 194 DEMIs were identified (Figure 2A,B). To identify miRNAs associated with low-temperature responses, the DEMIs among four comparisons: Se/S0, Sl/S0, Re/R0, and Rl/R0 were analyzed using Venn diagrams (Figure 2B). A total of 40 DEMIs appeared in two comparisons (Re/R0 and Rl/R0), while 16 DEMIs appeared in both Se/S0 and Sl/S0 (Figure 2B). This suggests that more miRNAs are involved in low-temperature responses in Yongning red rice. Nineteen miRNAs exhibited opposite expression patterns in Yongning red rice and B3 plants after low-temperature treatment (Figure 3). Among them, miR1437b-5P, miR166c-5P, miR1861h, miR395y, miR396d, and miR396h displayed expression trends similar to those of Dongxiang common wild rice under low-temperature treatment [19], indicating that these miRNAs are likely to be involved in the defense response of rice to cold stress.

miR156, miR166, miR167_1, and miR395 have been reported to play roles in plant defense against abiotic stress and diseases. For example, miR156 participates in the regulation of drought stress by targeting *StSPL9* in potato (*Solanum tuberosum* L.) [42]. miR166 promotes thermotolerance in *Arabidopsis* by regulating the expression of *Heat stress transcription factors* (*HSFA1*) [43]. miR395 is involved in the regulation of immunity in rice and the response to sulfate stress in *Brassica juncea* [36,44]. miR167_1 induces salicylic acid expression and stomatal closure by regulating target genes *AUXIN RESPONSE FACTOR 6* (*ARF6*) and *ARF8*, positively modulating *Arabidopsis* defense against *Pseudomonas syringae* [33]. Members of the miR156, miR166, miR167_1, and miR395 families were upregulated in Yongning red rice and downregulated in the B3 plants (Figure 3), suggesting their involvement in rice’s low-temperature response. There are also research reports that miR166 can affect nutrient absorption, photosynthesis, and grain quality in rice [31]. miR167_1 negatively regulates the expression of auxin (IAA) under high osmotic stress, influencing *Arabidopsis* root architecture [45]. These reports suggest miRNAs might indirectly participate in the stress response to low temperatures by altering metabolic processes and hormone regulation.

To identify low-temperature resistance-related genes, RNA sequencing was carried out in Yongning red rice and B3 under low-temperature treatment. Transcriptome analysis revealed significant differences in the response of Yongning red rice and B3 to low-temperature treatment. Compared to B3 plants, Yongning red rice exhibited a stronger defense response to low temperature at both early and late stages, with more upregulated DEMs (fold change ≥ 2) detected in Yongning red rice. Furthermore, upregulated DEMs were significantly more abundant than downregulated ones in Yongning red rice, indicating a stronger activation of cold-responsive genes.

To explore the defense mechanism of Yongning red rice against cold stress, DEMs during the early and late stages of low-temperature treatment in both varieties were analyzed. A total of 218 DEMs were oppositely expressed, with most being upregulated in Yongning red rice, suggesting that these DEMs positively regulate rice’s resistance to low temperatures. Ultimately, 21 genes were selected as potential candidates for participation in low-temperature defense (Table 2). Most of these genes were upregulated in Yongning red rice and downregulated in B3 plants after low-temperature treatment, with the exception of *LOC-Os07g45410*, highlighting their crucial role in the rice’s cold stress response. The gene *GRAIN INCOMPLETE FILLING 1* (*GIF1*), involved in sugar homeostasis mediated by cell wall invertase, plays an essential role in constitutive and induced defense responses [46]. Non-specific lipid transfer proteins (nsLTPs) contribute to various biological processes, including disease resistance [47,48,49,50,51], stress resistance [52,53,54,55,56,57], the regulation of germination and grain weight [58], and the formation of epidermal cutin and wax [59,60]. The EXPA gene family is also known to be vital in abiotic stresses, growth, and development [61,62,63,64].

Integrated expression analyses of miRNA and mRNA help identify functional miRNA–mRNA pairs related to rice resistance to cold stress. In this study, 82 specific DEMIs in Yongning red rice (Figure 2B) and 19 oppositely expressed miRNAs (Figure 3) corresponding to 416 target genes were identified under low-temperature treatment (Table S3). However, only 38 miRNAs corresponding to 39 target genes were potentially associated with rice’s low-temperature response (Table 3). For instance, miR169, known to negatively regulate immunity against *Magnaporthe oryzae* (rice blast fungus) [39], exhibited opposite expression patterns in Yongning red rice and B3 plants after low-temperature treatment (Figure 3). However, its target genes showed a similar expression pattern in both plant types, suggesting miR169 and its targets are not directly involved in cold stress response. This discrepancy could be attributed to several factors: (1) most target genes exhibited similar expression trends in both Yongning red rice and B3 plants after cold treatment, (2) the plant defense response to low temperature involves both systemic and local reactions, and many targets may not be expressed under these conditions, and (3) the criteria used for identifying DEMIs and DEMs may have overlooked key interactions.

## 5. Conclusions

In this study, we constructed 18 libraries of Yongning red rice and B3 plants before and after low-temperature treatment. These libraries were sequenced to identify mRNAs and miRNAs associated with cold resistance. Key miRNAs from families such as miR1437, miR156, miR166, miR1861, miR395, and miR396_2 were identified as playing significant roles in the rice’s resistance to low temperatures. DEMs involved in cold stress resistance were found to be related to plant cell wall modification, cellular processes, chitin response, and regulation of plant hypersensitive responses. Additionally, 38 miRNAs corresponding to 39 target genes were identified as potential miRNA–mRNA pairs linked to cold stress resistance. The integrated analysis of miRNAs and cold-responsive genes in rice provides a foundation for further research on the functions of miRNAs and their targets in low-temperature stress response, establishing a molecular basis for future studies on how plants adapt to cold environments.

## Figures and Tables

**Table 1:** DEMs between the Yongning red rice and B3 plants in response to low-temperature treatment.

Comparison	FC> 2		FC > 5		Total
	Down	Up	Down	Up	
Se/S0	3860	3614	990	1474	7474
Sl/S0	5088	4915	1892	2132	10,003
Re/R0	3493	4436	696	2338	7929
Rl/R0	4969	5623	1746	3177	10,592

**Table 2:** Candidate genes related to low-temperature resistance exhibiting an opposite expression in the early and late stages of low-temperature treatment in two rice materials.

#ID	Fold Change (log2)				Description	GO Term (BP)	GO Term (CC)
	Re/R0	Rl/R0	Se/S0	Sl/S0			
LOC_Os07g45410	−1.4679	−1.7799	3.5761	4.2068	Putative polyprotein	cellular process	cell part
LOC_Os01g05010	1.3993	1.1943	−1.3718	−1.2266	Hypothetical protein OsI_00351	single-organism cellular process	intracellular organelle part
LOC_Os01g12640	2.4200	2.9051	−2.2719	−2.4547	Probable membrane-associated kinase regulator 2	single-organism cellular process	nucleus
LOC_Os01g31800	2.4096	1.3732	−1.8766	−2.1555	Similar to Histone H2A	cellular process	nucleus
LOC_Os01g60740	7.3542	6.3104	−2.7176	−3.1318	Non-specific lipid-transfer protein 1	single-organism process	plasma membrane
LOC_Os02g33780	1.6491	1.3666	−1.3508	−1.7150	Uncharacterized abhydrolase domain-containing protein DDB_G0269086	cellular process	nucleus
LOC_Os03g04970	1.4419	1.2896	−1.2938	−2.1664	Chaperonin CPN60-1, mitochondrial	response to cadmium ion	plasma membrane
LOC_Os03g06670	4.2671	3.7827	−1.1857	−1.4218	Probable histone H2A variant 1	defense response to bacterium	nucleus
LOC_Os03g21820	2.4730	3.1456	−3.1503	−2.3354	Expansin OsEXPA6	plant-type cell wall modification	plasmodesma
LOC_Os04g02530	3.4447	3.2431	−4.3337	−3.7617	Hypothetical protein	\	cell part
LOC_Os04g33740	5.0839	4.8347	−3.0304	−2.8914	Cell-wall invertase, Carbon partitioning during early grain filling, Regulation of endosperm development	plant-type cell wall modification	plasma membrane
LOC_Os05g07820	3.6695	2.9440	−2.5463	−3.1852	Hypothetical protein OsI_18626	defense response to bacterium	plasma membrane
LOC_Os06g02900	4.0923	2.5764	−2.5671	−3.1472	Protein ASPARTIC PROTEASE IN GUARD CELL 2	response to stress	plasma membrane
LOC_Os06g49360	1.1686	1.0605	−1.6312	−2.3403	NBS-LRR disease resistance protein, putative, expressed	response to stress	cell part
LOC_Os08g39330	4.1966	2.3521	−2.1104	−3.1135	Hypothetical protein	single-organism process	cell part
LOC_Os08g40690	3.3571	2.2450	−1.1110	−2.7002	Xylanase inhibitor protein 1-like	metabolic process	cell part
LOC_Os08g41880	2.1007	1.8180	−1.0147	−1.7893	Nucleotide pyrophosphatase/phosphodiesterase	response to stress	plasmodesma
LOC_Os10g25450	4.2100	4.0353	−1.8408	−2.1186	Subtilisin-like protease SBT1.8	biological regulation	plasma membrane
LOC_Os12g05120	3.1791	2.4783	−1.7109	−2.1749	Leucine-rich repeat receptor-like protein kinase PXC1 isoform X1	cell wall organization	plasma membrane
LOC_Os12g38140	4.0395	3.6046	−2.0869	−2.8372	Uncharacterized protein LOC9267984	cellular process	nucleus
Oryza_sativa_newGene_1709	1.0610	1.1701	−1.2478	−1.1633	\	\	\

**Table 3:** The miRNA–mRNA interactions related to plant low-temperature response.

#ID	Fold Change (log2)				#ID	Fold Change (log2)				Description
	Se/S0	Sl/S0	Re/R0	Rl/R0		Se/S0	Sl/S0	Re/R0	Rl/R0	
miR1425-3p	0.0385	−0.2230	−0.1104	−0.9335	LOC_Os05g50654	--	0.8852	1.3105	2.2248	Mitochondrial import receptor subunit TOM7-1, putative, expressed
miR1437b-5p	0.2693	0.8142	−0.2584	−4.2600	LOC_Os01g08150	−0.1401	−0.4229	6.5867	6.1776	Uncharacterized protein LOC4326223
miR167a-5p	−0.2121	−0.6547	−0.1631	−1.0022	LOC_Os06g15810	−0.9276	−0.3215	0.3652	0.9685	Integral membrane protein, putative, expressed
miR167b	−0.1825	−0.6602	−0.1181	−1.0064						
miR167c-5p	−0.2121	−0.6547	−0.1631	−1.0022						
					LOC_Os08g04840	1.8926	2.8602	−0.3723	0.4304	MYB family transcription factor, putative, expressed
miR167h-3p	−0.8602	−1.3031	0.1685	0.4566	LOC_Os04g35610	0.9768	0.2621			Pentatricopeptide repeat-containing protein At2g13600
					LOC_Os08g38600	1.1403	1.9334			RING-type E3 ubiquitin ligase RGLG6
					LOC_Os03g14915	0.0898	0.4955	−2.4531	−2.5449	Tyrosine-specific transport protein 2 isoform X2
miR1861k	−0.1467	1.0855	2.8249	2.9707	LOC_Os01g63810	−0.0641	0.5740	−1.0251	−0.8952	Starch binding domain containing protein, putative, expressed
miR1861e	−0.1463	1.0859	2.8248	2.9707						
miR1861m	−0.1469	1.0861	2.8246	2.9702						
miR395y	−0.2012	−0.4800	1.2076	2.4779	LOC_Os12g38380	0.1141	−1.1143	−0.3338	−0.8822	Cleavage stimulation factor subunit 77 isoform X2
miR396a-5p	−0.0081	−0.6248	−0.5344	−1.0779	LOC_Os06g17900	−1.0074	−2.2897	1.1187	0.6906	Disease resistance protein RPM1, putative, expressed
miR396b-5p	−0.0081	−0.6248	−0.5369	−1.0804	LOC_Os06g28000	−0.9280	−1.1340	4.8473	5.0674	Carboxyl-terminal peptidase, putative, expressed
miR396h					LOC_Os02g45570	−2.0189				Growth-regulating factor 10 isoform X1
miR396g	0.2577	0.3042	−1.4890	−1.5029	LOC_Os06g02560	−0.2435	0.5606	1.1271	1.6195	Growth-regulating factor 5 isoform X1
miR396d										
miR3979-5p	−0.4130	−0.9323	1.0833	−0.2807	LOC_Os04g57220	0.7600	1.0155	−0.4384	0.5903	Ubiquitin-conjugating enzyme E2-17 kDa
					LOC_Os03g13460	1.0801	0.5897	−0.1653	0.3637	65 kDa microtubule-associated protein 6
miR5337a	0.5453	0.8813	3.9540	4.5778	LOC_Os03g06139	0.8186	1.1409	−1.0093	−0.0761	ABC transporter G family member 22 isoform X1
miR6245b-5p	−0.9684	−0.2557	2.8338	1.7829	LOC_Os11g47570	1.4791	1.2651			Xylanase inhibitor protein 2-like
novel_miR_102	−0.2667	0.2692	−0.7629	−0.1004	LOC_Os04g52880	−0.8309	−0.8725	4.5114	4.7659	Similar to H0714H04.3 protein
novel_miR_134	−0.2696	0.2566	−0.8044	−0.1253						
novel_miR_153	−0.2665	0.2695	−0.7629	−0.1004						
novel_miR_196	−0.2696	0.2566	−0.8044	−0.1253						
novel_miR_201	−0.2696	0.2566	−0.8044	−0.1253						
novel_miR_67	−0.4573	0.2371	−0.8884	−0.0795						
novel_miR_109	0.4099	−0.6760	−0.6026	−1.3629	LOC_Os08g36490	−0.6892	−1.0994	2.2559	2.1977	Kinetochore protein NDC80 homolog
					LOC_Os02g36890	−0.2098	0.0709	8.8909	9.4221	Myb-related protein Zm38
					LOC_Os04g52880	−0.8309	−0.8726	4.5114	4.7659	Similar to H0714H04.3 protein
novel_miR_114	0.6222	0.0161	−1.0781	−0.1472	LOC_Os11g03720	−3.2534	−2.8759			Similar to H0114G12.9 protein
					LOC_Os01g19694	−0.3074	−0.7558	4.8059		Homeobox protein knotted-1-like 1
novel_miR_119	0.0527	−0.1977	−1.2309	−1.6439	LOC_Os04g46620	0.0172	−0.0250	1.7292	1.6687	T-complex protein 1 subunit α
novel_miR_187	−0.0523	−0.4546	−1.0137	−1.3382	LOC_Os09g31170	−0.2716	−0.3146	1.0175	0.5548	Uncharacterized protein LOC4347421
					LOC_Os02g32615	−0.2060	0.2797	−1.5073	−0.7702	Expressed protein
novel_miR_120	0.2588	0.8452	2.3519	1.5828	LOC_Os09g13920	0.5713	0.7488	−1.4987	−1.0764	Uncharacterized protein LOC4346684
novel_miR_212	0.2586	0.8452	2.3516	1.5821	LOC_Os02g35820	1.2164	1.3602	−1.0794	−0.7511	Uncharacterized protein LOC4329708
					LOC_Os07g41420	1.5409	2.0942	−1.0222	−0.1276	Unknown protein
novel_miR_142	−0.2254	−0.4847	−0.9155	−0.8310	LOC_Os01g69290	0.2117	−0.0376	4.0603	4.0132	Antifreeze glycoprotein, putative, expressed
novel_miR_150	−0.6474	−1.0517	1.1072	0.4655	LOC_Os01g63810	−0.0641	0.5741	−1.0251	−0.8952	Starch binding domain containing protein, putative, expressed
novel_miR_180	0.0712	−0.1368	−0.6287	−0.8190	LOC_Os05g49040	−1.4883	0.1513	3.3286	4.4992	Cytochrome b561/ferric reductase transmembrane domain containing protein.
novel_miR_237	−0.2012	0.3890	−0.8272	−0.2583	LOC_Os04g52880	−0.8309	−0.8725	4.5114	4.7659	Similar to H0714H04.3 protein
novel_miR_5	0.4892	0.9472	1.3040	1.1625	LOC_Os03g53540	0.2154	−0.7403	−4.4586	−4.1983	Uncharacterized protein LOC4334093
					LOC_Os06g22560			−3.0613	−4.0183	Putative phosphoribosylglycinamide formyltransferase, chloroplast precursor
					LOC_Os09g13920	0.5713	0.7488	−1.4987	−1.0764	Uncharacterized protein LOC4346684
					LOC_Os11g35040			−1.2576	−0.8390	Probable aminotransferase TAT2
					LOC_Os10g31330	0.8122	0.5856	−1.1464	−1.2935	Glycine-rich cell wall structural protein 2
					LOC_Os10g01470	0.4421	−0.2362	−0.9824	−1.1884	Homeobox-leucine zipper protein HOX15
					LOC_Os03g07190	0.3451	1.5299	−0.9459	−1.0435	Uncharacterized protein LOC4331752
novel_miR_52	−0.3276	0.3383	−0.8528	−0.4219	LOC_Os03g60750	−0.1889	−0.1570	1.3812	0.9981	Ribosomal RNA large subunit methyltransferase J, putative, expressed
novel_miR_53	0.2661	1.3680	−1.1105	−0.8701	LOC_Os01g48010	−1.7158	−1.1017			Hypothetical protein OsI_03210
novel_miR_225	0.2652	1.3673	−1.1107	−0.8701						

## Data Availability

The data presented in this study are available on request from the first author Fan Luo. The data are not publicly available due to privacy restrictions.

## Supplementary Materials

The following supporting information can be downloaded at: https://www.mdpi.com/article/10.3390/genes16010038/s1, Figure S1: Heatmap of DEMIs in Yongning red rice and B3 after low-temperature treatment; Figure S2: GO (Gene Ontology) analysis of DEMs in Yongning red rice and B3 after low-temperature treatment; Table S1: Primers used in qRT-PCR; Table S2: Summary of small RNA sequences data; Table S3: Summary of mRNA expression libraries; Table S4: The miRNA-mRNA pairs related to the resistance of rice to low temperatures.
