# Supplementary figures and images for: Integrative Transcriptomic and Small RNA Analysis Uncovers Key Genes for Cold Resistance in Rice
Source: Genes (Basel). 2024 Dec 29;16(1):38. doi: 10.3390/genes16010038 (PMC11765247; doi:10.3390/genes16010038)

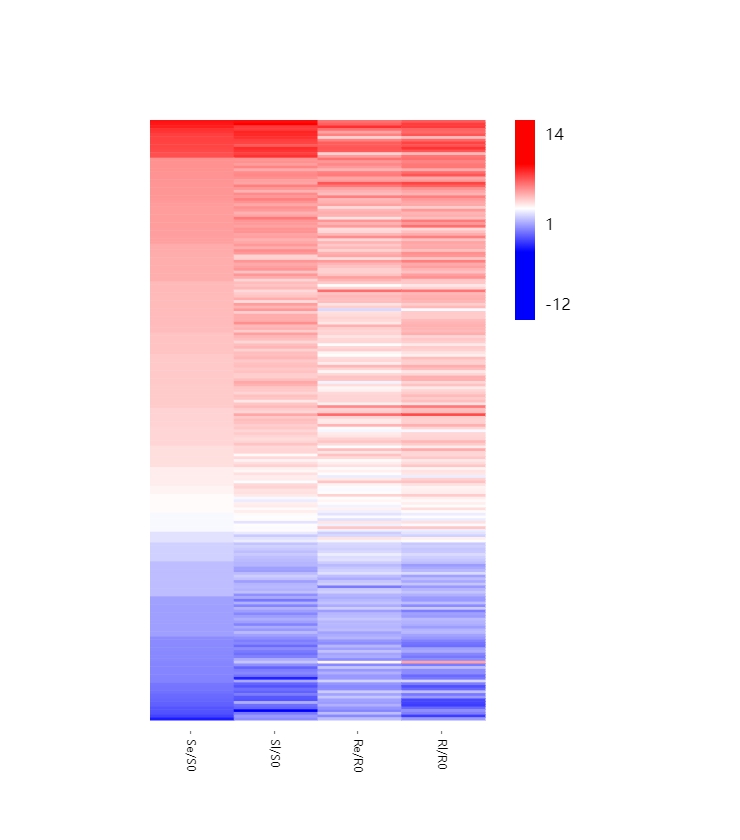

Supplement: Supplementary file 1 [file genes-16-00038-s001.zip › Figure S1.jpg]

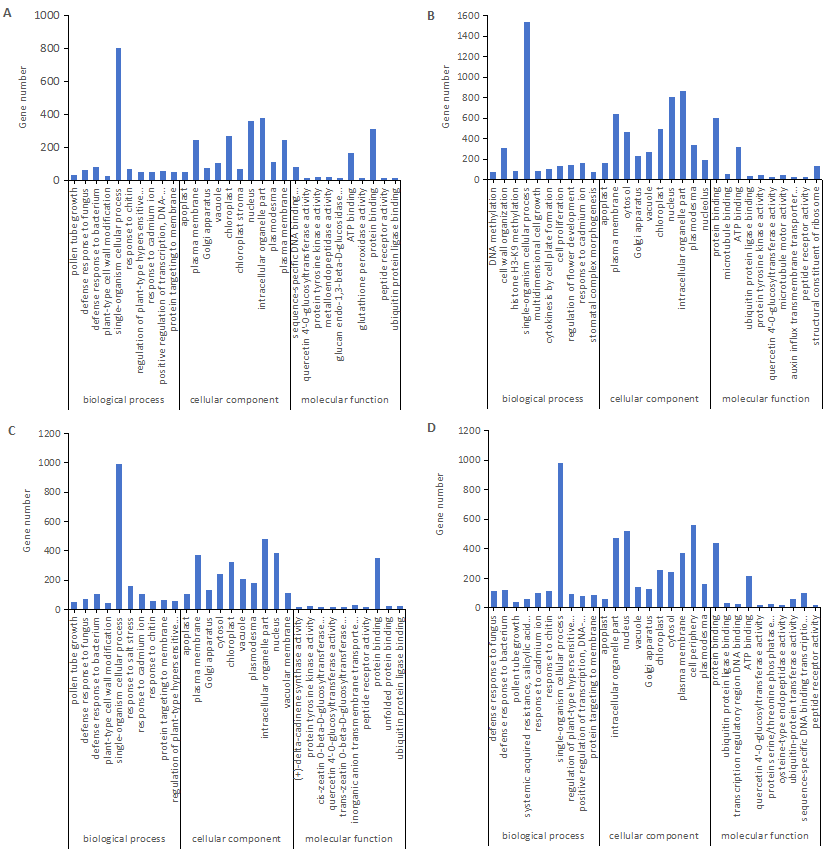

Supplement: Supplementary file 1 [file genes-16-00038-s001.zip › Figure S2.jpg]
